# Comparing the effectiveness and cost-effectiveness of alternative type 2 diabetes monitoring intervals in resource limited settings

**DOI:** 10.1093/heapol/czae072

**Published:** 2024-08-03

**Authors:** Elton Mukonda, Maia Lesosky, Siphesihle Sithole, Diederick J van der Westhuizen, Jody A Rusch, Naomi S Levitt, Bronwyn Myers, Susan Cleary

**Affiliations:** Division of Epidemiology & Biostatistics, School of Public Health, University of Cape Town, Anzio Road, Cape Town 7925, South Africa; National Heart and Lung Institute, Imperial College London, 1B Manresa Road, London SW3 6LR, United Kingdom; Division of Epidemiology & Biostatistics, School of Public Health, University of Cape Town, Anzio Road, Cape Town 7925, South Africa; Division of Chemical Pathology, Department of Pathology, University of Cape Town, Anzio Road, Observatory, Cape Town 7925, South Africa; National Health Laboratory Service, Groote Schuur Hospital, Main Road, Observatory, Cape Town 7935, South Africa; Division of Chemical Pathology, Department of Pathology, University of Cape Town, Anzio Road, Observatory, Cape Town 7925, South Africa; National Health Laboratory Service, Groote Schuur Hospital, Main Road, Observatory, Cape Town 7935, South Africa; Chronic Disease Initiative for Africa, Department of Medicine, University of Cape Town, Observatory, Cape Town 7925, South Africa; Curtin enAble Institute, Faculty of Health Sciences, Curtin University, GPO Box U1987, Perth WA 6845, Australia; Mental Health, Alcohol, Substance Use and Tobacco Research Unit, South African Medical Research Council, Francie van Zijl Drive, Parowvallei, Cape Town 7500, South Africa; Department of Psychiatry and Mental Health, University of Cape Town, Anzio Road, Observatory, Cape Town 7925, South Africa; Health Economics Unit, School of Public Health, University of Cape Town, Anzio Road, Observatory, Cape Town 7925, South Africa

**Keywords:** Type-2 diabetes, routine monitoring, low- and middle-income countries, cost-effectiveness

## Abstract

Type 2 diabetes (T2D) represents a growing disease burden in South Africa. While glycated haemoglobin (HbA1c) testing is the gold standard for long-term blood glucose management, recommendations for HbA1c monitoring frequency are based on expert opinion. This study investigates the effectiveness and cost-effectiveness of alternative HbA1c monitoring intervals in the management of T2D. A Markov model with three health states (HbA1c <7%, HbA1c ≥ 7%, Dead) was used to estimate lifetime costs and quality-adjusted life years (QALYs) of alternative HbA1c monitoring intervals among patients with T2D, using a provider’s perspective and a 3% discount rate. HbA1c monitoring strategies (three-monthly, four-monthly, six-monthly and annual tests) were evaluated with respect to the incremental cost-effectiveness ratio (ICER) assessing each comparator against a less costly, undominated alternative. The scope of costs included the direct medical costs of managing diabetes. Transition probabilities were obtained from routinely collected public sector HbA1c data, while health service utilization and health-related-quality-of-life (HRQoL) data were obtained from a local cluster randomized controlled trial. Other parameters were obtained from published studies. Robustness of findings was evaluated using one-way and probabilistic sensitivity analyses. A South African indicative cost-effectiveness threshold of USD2665 was adopted. Annual and lifetime costs of managing diabetes increased with HbA1c monitoring, while increased monitoring provides higher QALYs and life years. For the overall cohort, the ICER for six-monthly vs annual monitoring was cost-effective (USD23 22.37 per QALY gained), whereas the ICER of moving from six-monthly to three-monthly monitoring was not cost effective (USD6437.79 per QALY gained). The ICER for four-monthly vs six-monthly monitoring was extended dominated. The sensitivity analysis showed that the ICERs were most sensitive to health service utilization rates. While the factors influencing glycaemic control are multifactorial, six-monthly monitoring is potentially cost-effective while more frequent monitoring could further improve patient HrQoL.

Key messages

- The lack of consistent guidance regarding the optimal glycaeted haemoglobin (HbA1c) monitoring interval has key resource implications to low- and middle-income countries (LMICs) public health systems.
- To our knowledge this is the first study from Africa to assess the long-term effectiveness and cost-effectiveness of HbA1c monitoring in T2D.
- Using a Markov model, this study found that costs and quality-adjusted life years all increase with more frequent monitoring for both controlled and uncontrolled patients, while 6-monthly monitoring was cost-effective irrespective of whether patients meet targets for glycaemic control.
- While administering the HbA1C test is inexpensive in South Africa, this is not the case in most LMICs; hence, there is a need to balance maximizing the impact of monitoring to improve health outcomes while simultaneously reducing costs in other LMICs.

## Introduction

Type 2 diabetes (T2D) is an important non-communicable disease that is becoming a major healthcare concern in low- and middle-income countries (LMICs) from both a management and a health services affordability perspective (Tarride *et al*., 2010). T2D is associated with premature mortality and an increased risk of morbidity from complications, including cardiovascular disease (CVD), retinopathy, neuropathy and nephropathy (Rosenquist and Fox, 2018). T2D increases the economic burden for individuals and households as well as the overall health system (Seuring *et al*., 2015). In 2021, global diabetes-related health expenditure was estimated to be US$966 billion, representing a 316% increase from 2007 (International Diabetes Federation, 2021). As T2D prevalence is projected to markedly increase (International Diabetes Federation, 2021), particularly in LMICs, proportional increases in T2D health expenditure are also expected (Rosenquist and Fox, 2018; International Diabetes Federation, 2021). This could further destabilize LMIC health systems still reeling from infectious disease epidemics (Pastakia *et al*., 2017). A significant driver of T2D health expenditure is the management of T2D and its related complications, both microvascular and macrovascular.

Once diagnosed, managing T2D requires both effective treatment and ongoing monitoring, with the aim of achieving and maintaining glycaemic control, blood pressure control and lowering lipid levels (Glasziou *et al*., 2005). Glycated haemoglobin (HbA1c), which reflects blood glucose concentration over the previous 2–3 months, is regarded as the gold standard for long-term blood glucose management. Poor glycaemic control, generally defined as HBA1c ≥ 7%, is associated with increased risk of microvascular and macrovascular complications, as well as CVD and all-cause mortality (UK Prospective Diabetes Study (UKPDS) Group, 1998; Nathan, 2014). While the drivers of optimal glucose control are multifactorial, HbA1c monitoring can have an impact on glycaemic control through facilitating the improvement of treatment adherence, selection of treatments based on individual response, better titration of treatment and patients’ education about non-treatment factors (such as diet) that alter the condition’s control (Glasziou *et al*., 2005). However, due to a paucity of studies investigating the impact of monitoring frequency on clinical outcomes or cost-effectiveness, the recommendations for frequency of HbA1c monitoring are largely based on expert opinion (Mukonda and Lesosky, 2021). As a consequence, recommendations for HbA1c monitoring in T2D vary extensively (Mukonda and Lesosky, 2021).

Most guidelines in LMICs recommend monitoring HbA1c between one and four times a year depending on whether patients are meeting glycaemic control targets (Mukonda and Lesosky, 2021). In South Africa, monitoring guidelines are provided by the Society for Endocrinology, Metabolism and Diabetes of South Africa (SEMDSA), and the South African National Department of Health (NDOH) (Society for Endocrinology, Metabolism and Diabetes of South Africa, 2017; South African National Department of Health, 2020a). The SEMDSA guidelines recommend monitoring HbA1c at least every 6 months in patients with stable glycaemic control, and at 3-month intervals in patients not meeting targets in whom interventions have intensified. Primary care guidelines from NDOH, however, recommended annual HbA1c monitoring among patients with HbA1c < 8%, and 3three-monthly monitoring if HbA1c ≥ 8% or whenever there is a change in treatment (Society for Endocrinology, Metabolism and Diabetes of South Africa, 2017; South African National Department of Health, 2020a). The lack of consistent guidance regarding the optimal HbA1c monitoring interval has key resource implications for the South African public health system, where monitoring and treatment for T2D is free to patients at the point of care. Specifically, the lack of clear guidance could potentially lead to overuse of HBA1c monitoring, contributing to waste in healthcare and increased patient burden in diabetes management (McCoy *et al*., 2015; Ohde *et al*., 2018). In addition, the lack of definitive guidance can result in undertesting of HbA1c, leading to delayed or inadequate adjustments to treatment, ultimately leading to poor glycaemic control, as well as additional complications and associated costs.

With this background, the primary objective of this study was to investigate the optimal HbA1c monitoring interval by examining the long-term effectiveness and cost-effectiveness of alternative HbA1c monitoring intervals.

## Methods

This study presents a cost-utility analysis assessing the long-term effectiveness and cost-effectiveness of HbA1c monitoring strategies in South Africa. The monitoring strategies assessed are three-monthly tests (four tests per year), four-monthly tests (three tests per year), six-monthly tests (two tests per year) and annual (one test per year). The outcome measures are quality-adjusted life years (QALYs) and life years (LYs). The analysis is from the provider perspective. All costs were estimated in 2019 South African Rands and converted to US Dollars ($) using the average 2019 exchange rate of $1 = R14.45 (www.Oanda.com). Costs and benefits are calculated over a lifetime time horizon, with future costs and outcomes discounted at an annual rate of 3% with variation in sensitivity analysis. The incremental cost-effectiveness ratio (ICER) is estimated by arranging interventions from lowest to highest cost, and then comparing two adjacent non-dominated alternatives using the formula:


$$ICER = {\ }\frac{{Cos{t_{interval{\ }b}} - {\ }Cos{t_{interval{\ }a{\ }}}}}{{{\ }QAL{Y_{interval{\ }b}} - {\ }QAL{Y_{interval{\ }a}}}}\\[4pt]$$

Two types of dominance were assessed: (1) absolute dominance, where an intervention is more costly and less effective than another alternative and (2) extended dominance, where an intervention has a higher ICER than a previous less costly and non-dominated alternative. The remaining non-dominated alternatives are then assessed for potential cost-effectiveness by comparing to the indicative cost effectiveness threshold (CET) of $2662.00 per DALY averted, representing the marginal productivity of the South African health system (Edoka and Stacey, 2020). Given the lack of alternative threshold, it is assumed that a DALY-based threshold can be used to understand the value for money of QALY-based ICERs. The analysis is reported according to the Consolidated Health Economic Evaluation Reporting Standards statement (Supplementary material).

**Figure 1.**
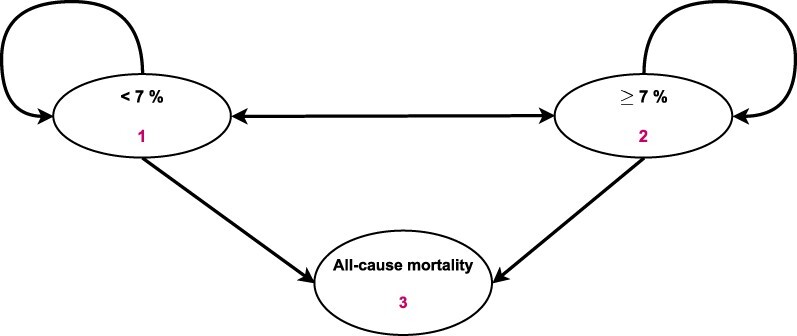
Markov Model for diabetes progression

A Markov model was developed and implemented in TreeAge Pro 2023 (TreeAge Software, Inc, Williamstown, Massachusetts, USA). The model simulates the progression of a hypothetical population of newly diagnosed patients with T2D, aged 40 years or older, using three Markov states: HbA1c <7%, HbA1c ≥ 7% and Dead (Figure 1). These states are parameterized for the associated costs and health outcomes of T2D patients at these levels of glycaemic control. The cycle length of the model is 1 year.

Several data sources are used in this study. Whenever possible, observational data collected in South Africa were used to parameterize the model and estimate costs. When observational data were unavailable, relevant, peer-reviewed published data were used.

Transition probabilities were estimated from individual-level data collected as part of routine clinical practice in the Western Cape, South Africa, by the National Health Laboratory Service (2021). Multi-state models, under Markov assumptions, were implemented to estimate the annual transition probabilities for the different monitoring strategies using a methodology described by Duff *et al*. (2018). The age-dependent all-cause mortality rates for the Western Cape population were derived from the Institute of Health Metrics and Evaluation Global Burden of Disease Study 2019 (GBD 2019 Demographics Collaborators, 2020). We assumed that people with well controlled T2D (HbA1c <7%) would have the same mortality rate as people without T2D (Rawshani *et al*., 2018). A relative risk of 1.30 [95% confidence interval (CI): 1.08, 1.55] was adopted for the effects of uncontrolled versus controlled diabetes on all-cause mortality (Navarro-Pérez *et al*., 2018).

The scope of provider costs included laboratory investigations and medicines as well as inpatient care, emergency room visits, outpatient visits and the management of diabetes-related complications. The average utilization of these resources was established for T2D patients with HbA1c <7% and HbA1c ≥ 7%. Patient costs were not considered in this study.

The cost of routine laboratory investigations was estimated by multiplying the unit costs of each test by the number of tests per year. Cost components were identified using the NDOH Standard Treatment Guidelines and Essential Medicines List (South African National Department of Health, 2020b). The tests include urine protein (1 per year), finger prick blood glucose (4 per year), serum creatinine (1 per year), serum potassium (one per year), HbA1c (variable: one to four times per year), one foot examination, and one retinal screening per year. Unit costs for the tests were obtained from the NHLS (National Health Laboratory Service, 2021).

Medication usage was obtained from NDOH Standard Treatment Guidelines and Essential Medicines List together with relevant literature on frequencies of use. The cost of treatment/medication was estimated by multiplying the unit costs of each type of medication and the amount required annually. We also assumed that of all diagnosed T2D cases, 22% use one oral medication, 27% use two or more oral medications, 41% use a combination of oral medication and insulin, while 9% use insulin only (Pinchevsky *et al*., 2017). The unit costs were obtained from the Medicine Price Registry provided by the NDOH (2022).

For the costs of inpatient care, emergency and outpatient department visits, we established the average utilization for T2D patients with HbA1c <7% and HbA1c ≥ 7% using primary data from a cluster randomized controlled trial (cRT). The trial enrolled 632 patients receiving diabetes care in 24 public sector primary care clinics in the Western Cape province of South Africa (Myers *et al*., 2018; 2022). These utilization estimates were then multiplied by local unit costs using the Health Systems Trust District Health Barometer (12th Edition—2019/20) datafile (Massyn *et al*., 2020), which provides estimates of expenditure per primary care visit, per outpatient/emergency department visit and per inpatient day from South African public sector hospitals (providing care for approximately 80% of those living in South Africa).

Average costs related to the management of diabetes-related complications were obtained from the literature (Erzse *et al*., 2019), with the cost of complications among patients with poor glycaemic control being 1.28 times higher than those with good control (Dall *et al*., 2016). Complications included renal disease, diabetic eye diseases (cataracts, retinopathy), diabetic foot disorder (amputations) and diabetic heart disease (stroke, ischaemic heart disease) (Dall *et al*., 2016). Using the Consumer Price Index (Statistics South Africa, 2020a), costs have been adjusted to the same base-year (2019) for comparison.

Health-related-quality-of-life (HRQoL) was measured using the EuroQol Five-Dimension questionnaire (EQ-5D-3 L) (Rabin and de Charro, 2001), based on an analysis of the baseline responses from diabetes patients enrolled in the same local cRCT (Myers *et al*., 2018; 2022). Since South Africa has yet to establish a local EQ-5D-3 L value set, the use of appropriate international valuation algorithms is recommended by NDOH HTA guidance (National Department of Health, 2013). The local cRCT data were valued using the UK time trade-off scoring algorithm, where a HRQoL value of 1 represents full health, while a value of 0 was set for death (Devlin *et al*., 2020). Valuing our local data using the UK value set resulted in a HRQoL value for patients with HbA1c <7% of 0.76, whereas the value for HbA1c ≥ 7% was 0.71. The estimates obtained align with other studies on HRQoL for people living with diabetes in sub-Saharan Africa (Kalayou Haftu *et al*., 2022; Jackson *et al*., 2023).

To account for the potential differences in monitoring among patients with good glycaemic control compared to those with poor control, a subgroup analysis was conducted with the two subgroups. The subgroup with good glycaemic control represents the scenario where patients had HbA1c <7% at time 0, whereas the subgroup with poor glycaemic control represents the scenario where patients had HbA1c ≥ 7% at time 0. Transition probabilities for both subgroups were estimated separately and are provided in the supplementary material. The same monitoring intervals were assessed.

Simple sensitivity analyses were run across key variables to determine the effects of parameter uncertainties on model robustness. Where possible, ranges for sensitivity analysis were based on upper and lower confidence intervals or interquartile ranges. A 25% increase/decrease in the cost per inpatient day, cost per outpatient visit, cost of treatment/medication and the cost of complications was applied to examine the sensitivity of estimates to fluctuations in cost. A probabilistic sensitivity analysis (PSA) with 10 000 iterations explored the uncertainties in the model parameters by randomly sampling values from each parameter distribution simultaneously and running the model for that set of parameter values to calculate the resulting outcomes of interest for each strategy. We calculated the cost, QALYs and ICERs from this sample. Cost-effectiveness acceptability curves, representing the probability of each strategy being cost-effective across all iterations were also plotted.

## Results

**Figure 2.**
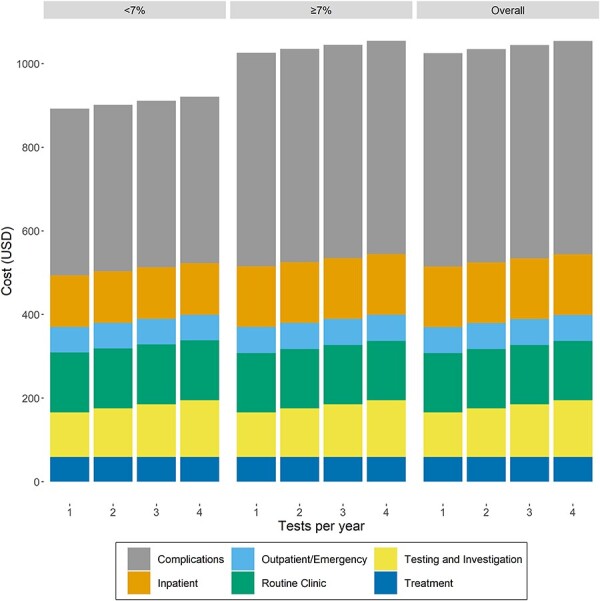
Average annual per patient cost of managing type 2 diabetes by glycaemic control status and number of HbA1c tests a year

Table 1 provides a summary of the variables used in the model, together with the ranges and distributions of variables used for sensitivity analyses. The cost per inpatient day was estimated at USD244 while the cost per clinic visit was USD39. The cost of routine management varies depending on the number of HbA1c tests administered per year, with the costs ranging from USD106.46 for 1 HbA1c test to USD135.40 for four HbA1c tests. The annual per patient cost of managing diabetes varies by the number of HbA1c tests a year and glycaemic control (Figure 2). Overall, the average annual direct medical cost of diabetes management ranges from USD1004.39 per patient for one HbA1c test a year, to USD1033.33 per patient for four HbA1c tests a year, based on our data where 84% of the trial population did not meet recommended targets for glycaemic control.

**Table 1.** Markov model input parameters: health resource utilization, HRQoL, costs and transition probabilities

Component	Base case value	PSA distribution	Source
Health resource utilization			
Mean inpatient days/year (SD) : HbA1C < 7	7 (10)	Gamma	Myers *et al*. (2022)
Mean inpatient days/year (SD) : HbA1C ≥ 7	9 (14)	Gamma	
Clinic visits/year: HbA1C < 7	4	Gamma	Myers *et al*. (2022)
Clinic visits/year: HbA1C ≥ 7	4	Gamma	Myers *et al*. (2022)
Mean emergency/outpatient department visits per year (SD) : HbA1C < 7	0.75 (2.56)	Gamma	Myers *et al*. (2022)
Mean emergency/outpatient department visits per year (SD) : HbA1C ≥ 7	0.77 (3.07)	Gamma	Myers *et al*. (2022)
HRQoL			
Mean HRQoL (SD) : HbA1C < 7	0.75 (0.35)	Beta	Myers *et al*. (2022)
Mean HRQoL (SD) : HbA1C ≥ 7	0.71 (0.34)	Beta	Myers *et al*. (2022)
Costs (USD)			
Cost per inpatient day	244.66	Gamma	Massyn *et al*. (2020)
Cost per clinic visit	38.97	Gamma	Massyn *et al*. (2020)
Cost per emergency/outpatient department visit	81.55	Gamma	Cunnama *et al*. (2016); Massyn *et al*. (2020)
Average cost of complications for patients with controlled T2D	398.53	Gamma	Erzse *et al*. (2019)
Average cost of complications for patients with uncontrolled T2D	510.12	Gamma	Dall *et al*. (2016), Erzse *et al*. (2019)
Average cost of treatment	59.31	Gamma	NDOH 2020b, Pinchevsky *et al*. (2017)
Testing/Investigation (USD)			
1 HbA1c test a year	106.46	Gamma	NDOH 2020b, National Health Laboratory Service (2021)
2 HbA1c test a year	116.11	Gamma	NDOH 2020b, National Health Laboratory Service (2021)
3 HbA1c test a year	125.75	Gamma	NDOH 2020b, National Health Laboratory Service (2021)
4 HbA1c test a year	135.40	Gamma	NDOH 2020b, National Health Laboratory Service (2021)
CET	2661.97		Edoka and Stacey (2020)
Transition probabilities Mean(Confidence Interval)			
Annual: HbA1C ≥ 7 to HbA1C < 7	0.17 (0.15;0.19)	Beta	National Health Laboratory Service (2021)
6-monthly: HbA1C ≥ 7 to HbA1C < 7	0.24 (0.22;0.27)	Beta	National Health Laboratory Service (2021)
4-monthly: HbA1C ≥ 7 to HbA1C < 7	0.26 (0.23;0.29)	Beta	National Health Laboratory Service (2021)
3-monthly: HbA1C ≥ 7 to HbA1C < 7	0.28 (0.24;0.30)	Beta	National Health Laboratory Service (2021)
Annual: HbA1C < 7 to HbA1C ≥ 7	0.58 (0.53;0.63)	Beta	National Health Laboratory Service (2021)
6-monthly: HbA1C < 7 to HbA1C ≥ 7	0.67 (0.62;0.71)	Beta	National Health Laboratory Service (2021)
4-monthly: HbA1C < 7 to HbA1C ≥ 7	0.69 (0.65;0.73)	Beta	National Health Laboratory Service (2021)
3-monthly HbA1C < 7 to HbA1C ≥ 7	0.69 (0.64;0.73)	Beta	National Health Laboratory Service (2021)
Initial probabilities for the Markov states			
Proportion with HbA1C < 7	0.16	-	Myers *et al*. (2022)
Proportion with HbA1C ≥ 7	0.84	-	Myers *et al*. (2022)

The discounted base case results are presented in Table 2, including the total cost, QALYs, and LYs gained for each strategy. The discounted lifetime cost of managing T2D is USD19 453.51 per patient for annual monitoring, USD19 577.15 for six-monthly monitoring, USD19 751.87 for four-monthly monitoring and USD19 913.16 per patient when monitoring HbA1c three-monthly. Similarly, annual is the least effective strategy (14.05 discounted QALYs and 19.53 LYs), followed by six-monthly (14.10 discounted QALYs and 19.57 LYs), four-monthly (14.12 discounted QALYs and 19.59 LYs) and three-monthly (14.16 discounted QALYs and 19.61 LYs). In terms of ICERs, six-monthly monitoring (USD2322.37 per QALY gained) is potentially cost effective, four-monthly monitoring is extended dominated and three-monthly monitoring (USD6437.79 per QALY gained) is unlikely to be cost-effective in comparison to the indicative CET. The same trend is observed for ICERs per LY gained, with ICERs of USD3091 for six-monthly monitoring and USD8400 for three-monthly monitoring. The subgroup analysis results are in line with the base case analysis although ICERs are considerably lower in those with controlled diabetes and higher in those with uncontrolled diabetes.

**Table 2.** Discounted base-case cost-effectiveness results for the frequency of monitoring HbA1c in a year: Costs (USD), QALY, LYs, ICERs and Net Monetary Benefit

Dominance	Strategy	Cost (USD)	Incremental costs (USD)	QALYs	Life Years	Incremental QALYs	Incremental LYs	ICER per QALY	ICER per LY	Net Monetary Benefit (QALY)	Net Monetary Benefit (LY)
Overall											
undominated	annual	19 453.51		14.05	19.53					17 947.17	32 535.35
undominated	6-monthly	19 577.15	123.64	14.1	19.57	0.05	0.05	2322.37	3 091	17 956.63	32 518.19
*ext. dominated	4-monthly	19 751.87	174.72	14.12	19.59	*	*	*	*	17 835.15	32 396.71
undominated	3-monthly	19 913.16	336	14.16	19.61	0.05	0.02	6437.79	8 400	17 780.34	32 288.66
Controlled diabetes											
undominated	annual	19 478.61		14.13	19.59					18 135.03	32 669.97
undominated	6-monthly	19 496.05	17.43	14.18	19.63	0.05	0.04	348.69	435.75	18 250.68	32 759.01
*ext. dominated	4-monthly	19 681.87	185.82	14.19	19.63	*	*	*	*	18 091.48	32 573.19
undominated	3-monthly	19 844.36	348.31	14.22	19.66	0.04	0.03	8869.24	11 610.33	18 008.85	32 490.56
Uncontrolled diabetes											
undominated	annual	19 462.43		14.02	19.51					17 858.39	32 473.19
undominated	6-monthly	19 583.93	121.5	14.1	19.57	0.08	0.06	1495.27	2025	17 949.85	32 511.41
*ext. dominated	4-monthly	19 765.21	181.28	14.11	19.58	*	*	*	*	17 795.19	32 356.75
undominated	3-monthly	19 926.18	342.25	14.14	19.6	0.04	0.03	7767.35	11 408.33	17 714.08	32 249.02

* Strategies are extended dominated.

**Figure 3.**
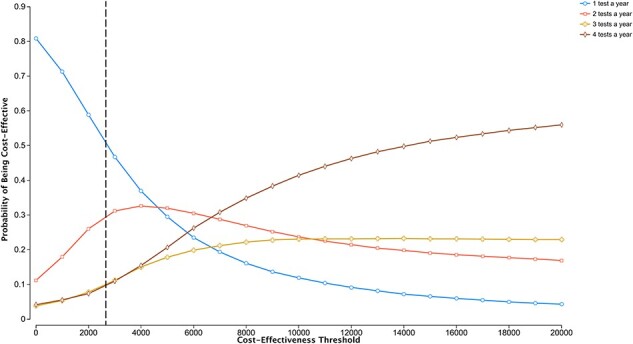
Cost effectiveness Acceptability Curves displaying the probability of each strategy being cost-effective across all simulations of the PSA over a range of cost-effectiveness thresholds. The black vertical line represents the South African CET

One-way sensitivity analyses were run on all variables presented in Table 1. The tornado diagrams (available in the supplementary material) summarize the results from the one-way sensitivity analyses that generated the largest changes to the ICER. All other analyses generated negligible changes in the ICER. The one-way sensitivity analyses suggested the ICER was most sensitive to health service utilization rates and less sensitive to the discount rate. The PSA revealed that testing annually (the least costly strategy) has a higher probability of being cost-effective for a CET lower than USD4500 compared with other testing frequencies, while testing three-monthly has a higher probability of being cost effective for a CET higher than USD6500 (Figure 3).

## Discussion

In this study, we investigated the cost and cost-effectiveness of alternative HbA1c monitoring frequencies as an intervention to improve glycaemic control in patients with T2D in South Africa. To our knowledge, this is the first such study conducted in Africa. Our results indicate that lifetime costs, QALYs and LYs all increase with more frequent monitoring. Using an indicative South African CET (Edoka and Stacey, 2020), 6-monthly monitoring (twice a year) is the most cost-effective strategy for the entire cohort as well as for subgroups of controlled/uncontrolled T2D patients. Of note, our analysis suggests that monitoring is more cost-effective in those with good glycaemic control. This does not mean that relatively higher investments should be made in the care of well-controlled patients. Instead the finding reflects the challenges of achieving glycaemic control in many of those living with diabetes, suggesting that monitoring should be complemented with additional interventions to improve glycaemic control in those with a higher HbA1c.

While some international sources are used, the data for this study are predominantly drawn from the South African public health sector, which serves approximately 80% of the population using a tax-based pre-payment mechanism. HbA1c monitoring is offered via a network of primary care clinics that are designed to be geographically accessible to all communities. In this system, no user fees are charged for primary care services and minimal means-tested user fees are charged for inpatient care. Despite this, patients may incur transport costs, lost income and other opportunity costs for time spent seeking care. This means that less frequent monitoring may be preferable from a patient perspective. Similarly, from a provider’s perspective, our results indicate that testing costs increase with frequency of monitoring. These patient opportunity costs and provider monitoring costs need to be balanced against the value of more frequent testing for informing clinical decisions and patient health promotion (Hirst *et al*., 2021).

There are a few studies from high-income countries that have investigated the impact of HbA1c monitoring as an intervention for achieving glycaemic control, but only one of these was an economic evaluation (Fu *et al*., 2012; Oke *et al*., 2012; Driskell *et al*., 2014; Wermeling *et al*., 2014; Duff *et al*., 2018; Ohde *et al*., 2018; Imai *et al*., 2021). Among the studies focused on patients who do not meet targets for glycaemic control (Fu *et al*., 2012; Driskell *et al*., 2014; Duff *et al*., 2018), there is consensus that three-monthly monitoring leads to greater reductions in HbA1c. Duff *et al*. (2018), however, found that testing two or three times a year was equally effective as four times per year for achieving glycaemic control among patients with sub-optimally controlled diabetes. In our study, we achieve similar results with more frequent monitoring, yielding marginally more QALYs and LYs. However, like Duff *et al*. (2018), we found relatively small differences in effectiveness between monitoring three-monthly and monitoring four- or six-monthly for patients with suboptimal disease control. Given these relatively small improvements in outcomes, six-monthly monitoring was most likely to be cost effective, while three-monthly monitoring was not cost-effective in comparison to an indicative South African CET (ref Edoka). Among studies focusing on patients with well-controlled diabetes (Oke *et al*., 2012; Wermeling *et al*., 2014; Ohde *et al*., 2018; Imai *et al*., 2021), longer monitoring intervals (six-monthly or annual) are often proposed. In their economic evaluation, Wermeling *et al*. (2014) sought to determine the effectiveness and the cost-effectiveness of three-monthly vs six-monthly monitoring among patients with well-controlled diabetes (defined as patients with HbA1c ≤58 mmol/mol or approximately ≤7.5%, systolic blood pressure ≤145 mmHg and total cholesterol ≤5.2 mmol/l). The authors found no difference in effectiveness between three-monthly and six-monthly monitoring, while six monthly monitoring was less costly.

When interpreting the results of this study for other LMIC settings, some care should be taken. For example, patients pay user fees in many LMIC settings (Asante *et al*., 2020) and such settings often employ centralized testing models that require patients with T2D to travel to hospitals and referral facilities in major urban centres to get an HbA1c test. The use of point-of-care (POC) HbA1c testing in primary care facilities can improve patient experience, reduce costs for the patient and potentially reduce patient burden at healthcare facilities (Al-Ansary *et al*., 2011; Schnell *et al*., 2017; Park and Pastakia, 2018; Hirst *et al*., 2021; Rosa *et al*., 2021).

In addition to evaluating the potential of HbA1c monitoring as an intervention, our findings also provide useful insights into the affordability of T2D management in South Africa. Approximately 2 million adults in South Africa access the public sector for their T2D care (International Diabetes Federation, 2021). Given the annual costs estimated in this study, the public sector budget impact amounts to USD1.7 billion, which is more than 10% of total government health expenditure (Statistics South Africa, 2020b; Williams *et al*., 2020). As such, the cost, cost-effectiveness and budget impact of alternative interventions for T2D treatment is an essential input to priority setting and maximizing value for money both in South Africa and in other LMICs where the burden of T2D is set to increase markedly in the coming decades. Second, the major cost drivers identified in our study and the study by Erzse *et al*. (2019), included managing complications, non-routine visits to healthcare facilities (inpatient, outpatient, emergency) and medications. With good primary care and early screening, it is possible to prevent or delay the onset of these complications—and reduce the number of hospitalizations and the length of stay in a facility, thereby lowering the annual cost of managing diabetes. Moreover, ramping up diabetes prevention may reduce the incidence of diabetes in South Africa, while targeted screening of high-risk individuals can potentially lower costs in future as many patients are only diagnosed with T2D at a late stage in disease progression (Aschner *et al*., 2021; Grundlingh *et al*., 2022).

Despite its importance, our study has limitations that warrant consideration. First, our study makes use of HRQoL and healthcare utilization data from the baseline assessment of 632 patients enrolled in a local cluster randomized trial. Given the focus of this trial on mental health interventions, it is possible that patients had relatively poorer health than the general population of T2D patients in South Africa. In addition, there are inherent limitations associated with using data from a cRT for this kind of modelling including potential for selection bias, limited generalizability as well as the impact of intra-cluster correlation (Donner and Klar, 2004). Despite this, the baseline assessment from Project Mind provides valuable insights into the care-seeking behaviour of patients with diabetes and co-occurring depression (which has a high prevalence among patients with T2D) and/or alcohol use disorder in the Western Cape, South Africa. Second, there is a possibility that both annual and lifetime costs are underestimated, as the study does not factor in costs related to mortality, lipid-lowering or blood pressure-lowering medications and the impact of other chronic disease comorbidities. Third, we conducted our analysis from the healthcare provider’s perspective even though conducting the study from a societal perspective may potentially provide a more nuanced perspective on the impact of HbA1c monitoring on both the healthcare provider and the patient with diabetes. Fourth, due to inherent limitations of the data available, this study was unable to conduct a detailed analysis of the distributional effects on different populations, including disadvantaged or priority populations. However, it is worth noting that the utilization and HRQoL data from the MIND study were collected from a disadvantaged population. Moreover, over 80% of the South African population uses the public health system, with the majority being uninsured and experiencing some degree of poverty. Lastly due to the complexity of diabetes, the use of a Markov model with only three states to model diabetes is a simplification. Other risk factors (e.g. ethnicity, duration of diabetes, smoking status), or other unmeasured confounders that may influence HbA1c progression were not accounted for in our model. While diabetes modelling would benefit more from patient-level simulation modelling that also incorporates the aforementioned risk factors, as well as the history of diabetes-related complications (Mukonda *et al*., 2021), the data needed to develop or validate such models are currently not available in South Africa and most other LMICs.

As current HbA1c monitoring recommendations are largely based on expert opinion or clinical consensus, our study not only corroborates the use of current monitoring guidelines but also proposes monitoring HbA1c twice a year as a cost-effective alternative to the least costly monitoring strategy (annual monitoring). More high-quality evidence, however, is needed to determine the optimal HbA1c monitoring strategy in a South African setting. Elwenspoek *et al*. (2020) suggest the use of randomized or cluster randomized controlled trials which include an economic evaluation to compare monitoring strategies. There is also a need to conduct similar studies for the frequency of lipid, and blood pressure measurement given their importance on the effective management of T2D. Overall, there is also a need to upscale national or sub-national level clinical and observational research on T2D interventions, such as the effectiveness and cost-effectiveness of HbA1c POC testing from a societal perspective in LMICs (Afroz *et al*., 2018; Masuku *et al*., 2022). This ensures that LMICs do not merely adopt interventions from HICs without assessing their cost-effectiveness as this may lead to a waste of already constrained resources. Moreover, these studies can be used to parameterize more suitable, locally relevant patient level T2D simulation models, or validate existing ones [48]. This study also highlights the need to investigate the long-term implications (financial and clinical) of different glycaemic control targets, particularly in the South African context.

## Conclusion

This analysis provides key evidence on the effectiveness and cost-effectiveness of varying intervals of T2D monitoring in South Africa. Overall, while monitoring six-monthly was found to be cost-effective in this study, monitoring three-monthly for patients with suboptimal glycaemic control ensures timely intervention to improve patient outcomes and reduce patient harms and the healthcare costs associated with these harms (Park and Pastakia, 2018). Among patients who meet targets for glycaemic control, six-monthly or annual monitoring reduces, albeit slightly, the treatment burden and healthcare costs. The high costs associated with T2D management highlight the need to improve T2D prevention, screening and diagnosis, as such efforts could result in cost savings in the long term.

## Supplementary Material

czae072_Supp

## Data Availability

De-identified patient data that support the findings of this study are not publicly available but may be obtained from the National Health Laboratory Service (NHLS). Restrictions apply to the availability of these data, which were used under license for this study. Data are available from the authors with the permission of NHLS.

## Reflexivity statement

The authors include three males and five females and span varying levels of seniority from Research Assistant to Professor. The first author (E.M.) has a background in statistics and population health, with a specific interest in HIV/AIDS and diabetes in South Africa. M.L. has extensive expertise across a range of topics in biostatistics and is involved a number of studies on respiratory and lung health in sub Saharan Africa, where she also worked for over 15 years. S.S. is the most junior contributor who, as an African female, is a part of an under-represented demographic in science. D.V.W., J.R., B.M. and N.L. have extensive experience in the medical field, particularly non-communicable diseases, while S.C.’s field of expertise is health systems and health economics.

Note on authorship: This study uses South Africa as a case study to address a problem that may affect other LMICs. As required, at least one of the authors is from an LMIC.
