# Supplementary material for: Comparing the effectiveness and cost-effectiveness of alternative type 2 diabetes monitoring intervals in resource limited settings
Source: Health Policy Plan. 2024 Aug 3;39(9):946–55. doi: 10.1093/heapol/czae072 (PMC11474914; doi:10.1093/heapol/czae072)
Supplement: czae072_Supp [file czae072_supp.zip › Cost_effectiveness_of_monitoring_strategies_HPP_Appendix (1).docx]

**Table S1 Annual cost of managing diabetes in the base case analysis, assuming 84% of the diabetic population is uncontrolled and 16% is controlled**

|  | **Controlled** | **Uncontrolled** | **Average** |
| --- | --- | --- | --- |
| **Monitoring Interval** | **USD** | **USD** | **USD** |
| Annual | 892.11 | 1,025.78 | 1,004.39 |
| 6-monthly | 902.76 | 1,035.43 | 1,014.20 |
| 4-monthly | 911.41 | 1,045.08 | 1,023.69 |
| 3-monthly | 921.06 | 1,054.72 | 1,033.33 |
| SEMDSA - 3-monthly /6-monthly | 902.76 | 1,054.72 | 1,030.40 |
| NDoH - 3-monthly / annual | 892.11 | 1,054.72 | 1,028.70 |


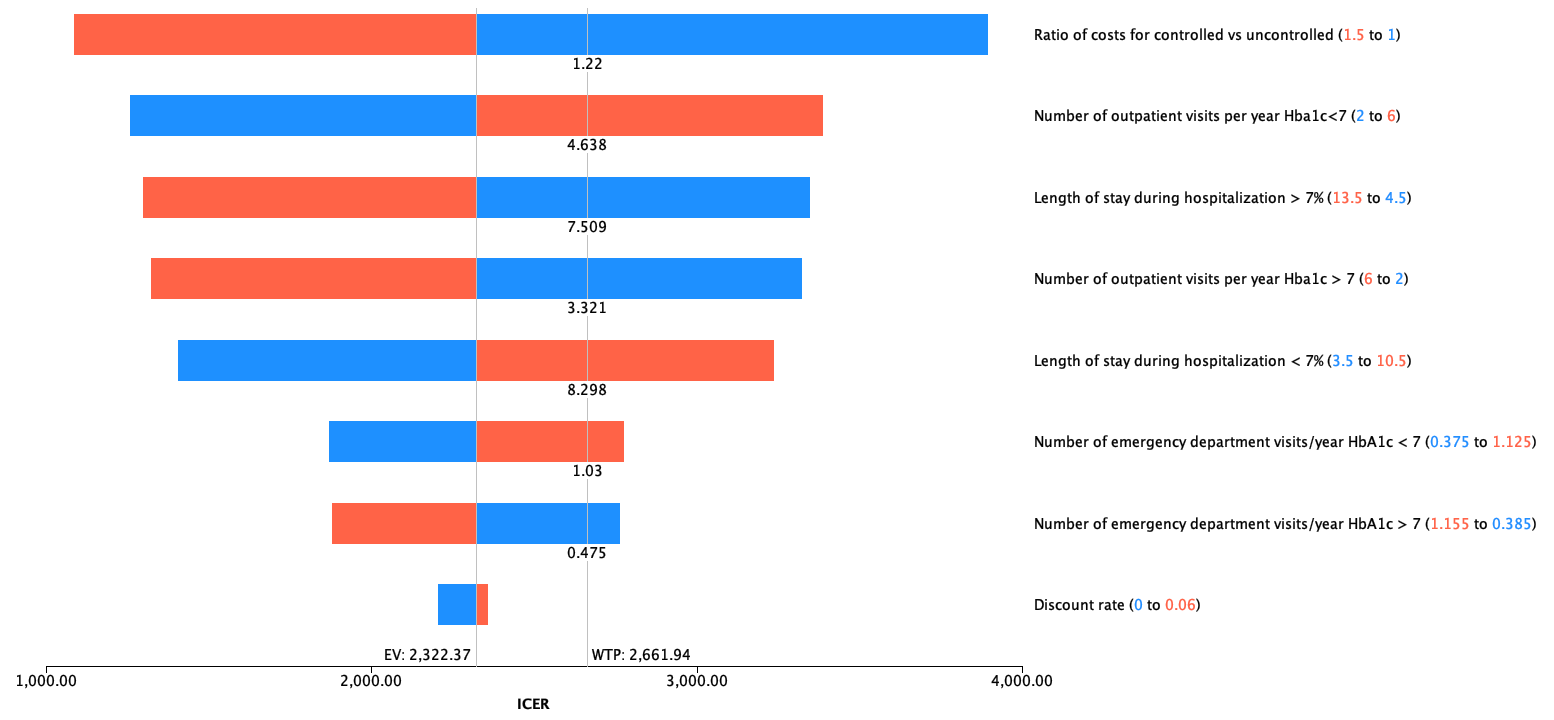


**Figure S1 Tornado diagram for one way sensitivity analysis of 6 monthly test vs annual testing. Expected Value (EV) of $2,332 on the x-axis represents the base case cost per QALY gained. ICER is the incremental cost‐effectiveness ratio and WTP is the willingness to pay.**


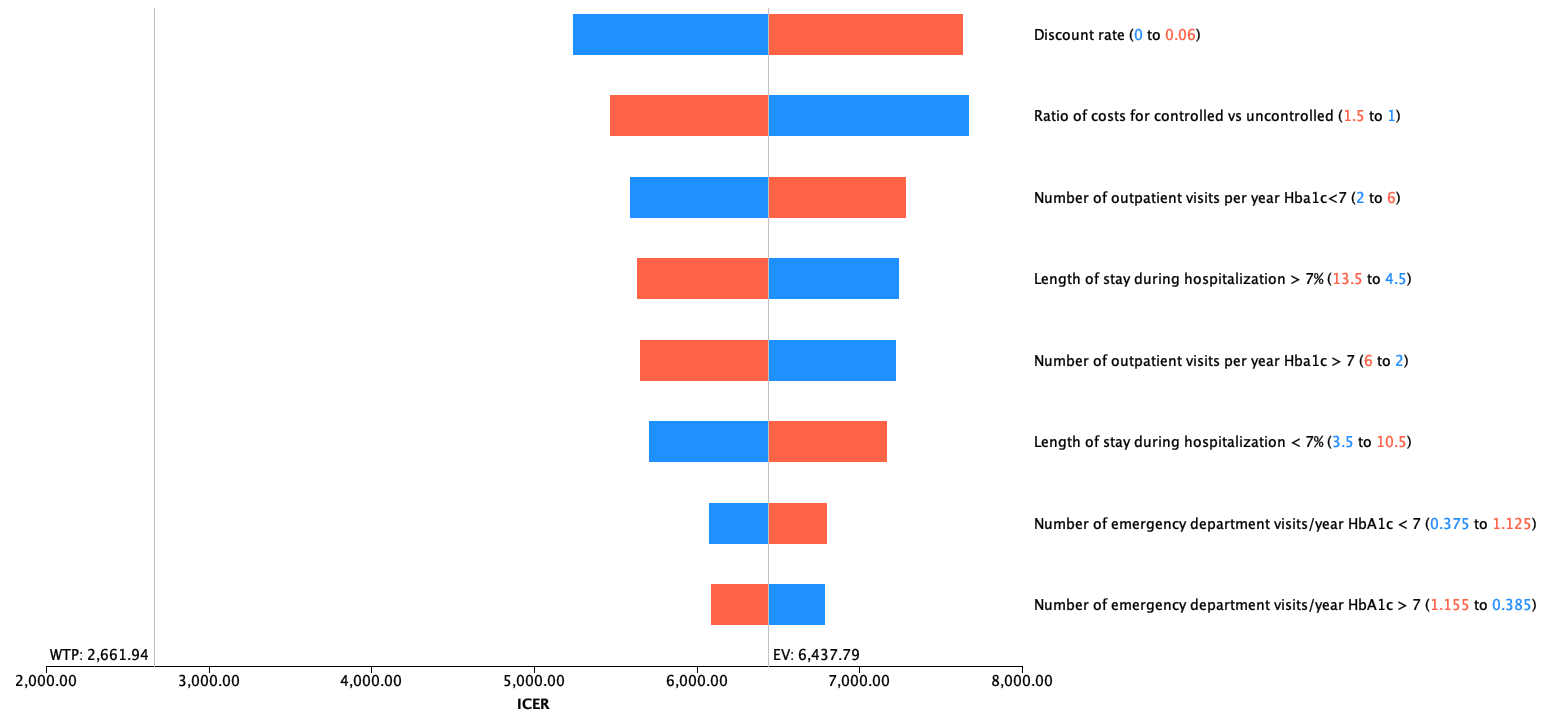


**Figure S2 Tornado diagram for one way sensitivity analysis of 3-monthly vs 6-monthly testing. Expected Value (EV) of $6,437.79 on the x-axis represents the base case cost per QALY gained. ICER is the incremental cost‐effectiveness ratio and WTP is the willingness to pay.**


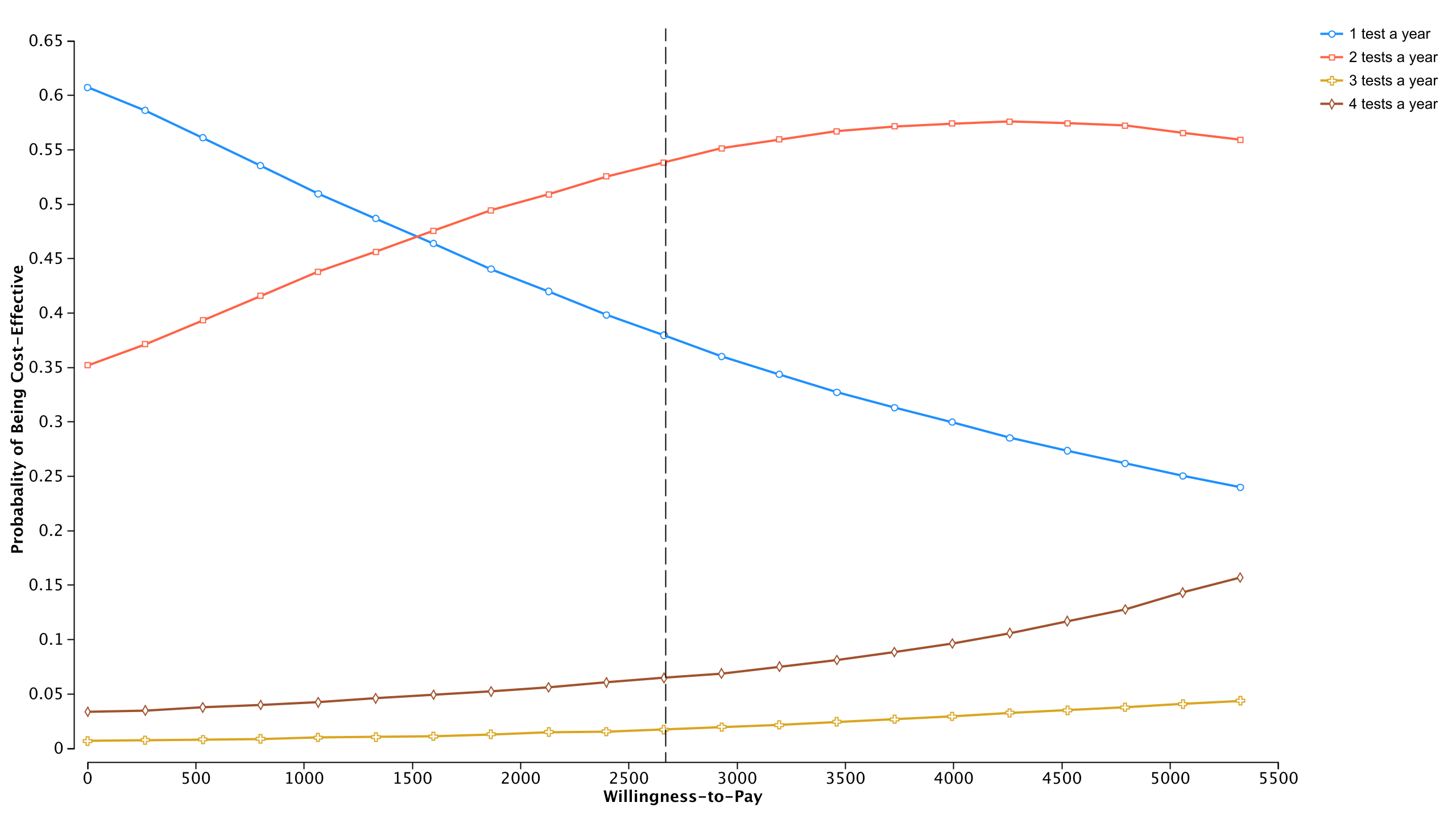


**Figure S3 Cost effectiveness Acceptability Curves displaying the probability of each strategy being cost-effective across all simulations of the PSA over a range of cost-effectiveness thresholds for the controlled subgroup. The black vertical line represents the South African cost-effectiveness threshold**


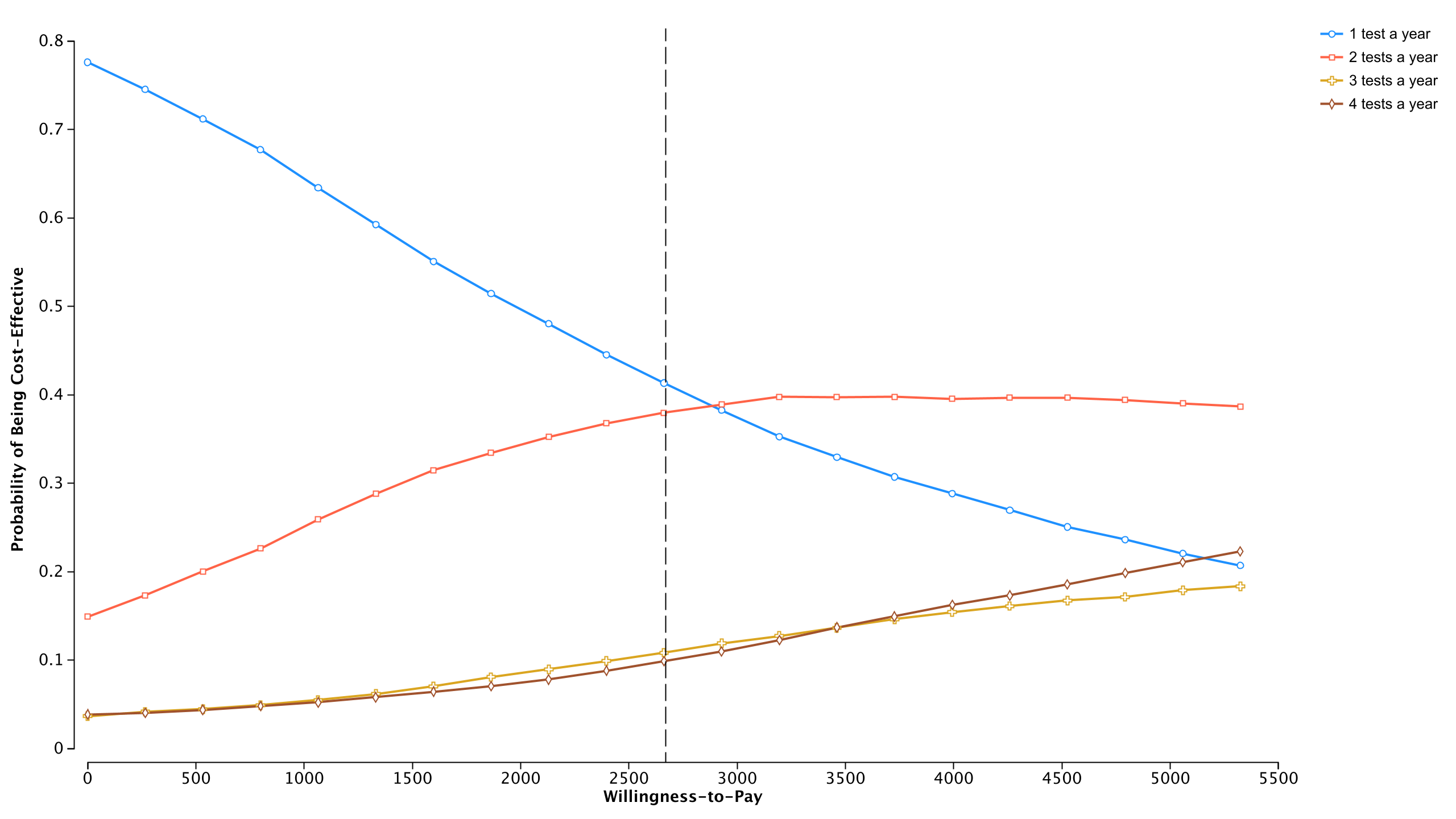


**Figure S4 Cost effectiveness Acceptability Curves displaying the probability of each strategy being cost-effective across all simulations of the PSA over a range of cost-effectiveness thresholds for the uncontrolled subgroup. The black vertical line represents the South African cost-effectiveness threshold**

CHEERS 2022 Checklist

|  | **Item** | **Guidance for Reporting** | **Reported in section** |
| --- | --- | --- | --- |
| **TITLE** | | |  |
| Title | 1 | Identify the study as an economic evaluation and specify the interventions being compared. | Title page |
| **ABSTRACT** | | |  |
| Abstract | 2 | Provide a structured summary that highlights context, key methods, results and alternative analyses. | Page 1 |
| **INTRODUCTION** | | |  |
| Background and objectives | 3 | Give the context for the study, the study question and its practical relevance for decision making in policy or practice. | Page 2  Page 3  Page 4 Line 1 - 3 |
| **METHODS** | | |  |
| Health economic  analysis plan | 4 | Indicate whether a health economic analysis plan was developed and where available. | NA |
| Study population | 5 | Describe characteristics of the study population (such as age range, demographics, socioeconomic, or clinical characteristics). | Page 5 Line 6 |
| Setting and location | 6 | Provide relevant contextual information that may influence findings. | Page 3 Line 9 – 20  Page 4 Line 23 – 24  Page 5 Line 15 – 21  Page 5 Line 15 – 21 |
| Comparators | 7 | Describe the interventions or strategies being compared and why chosen. | Page 4; Line 8 - Line 9 |
| Perspective | 8 | State the perspective(s) adopted by the study and why chosen. | Page 4; Line 10 |
| Time horizon | 9 | State the time horizon for the study and why appropriate. | Page 4; Line 12 |
| Discount rate | 10 | Report the discount rate(s) and reason chosen. | Page 4; Line 13 |
| Selection of outcomes | 11 | Describe what outcomes were used as the measure(s) of benefit(s) and harm(s). | Page 4; Line 9-10 |
| Measurement of outcomes | 12 | Describe how outcomes used to capture benefit(s) and harm(s) were measured. | Page 7; Line 14 - Line 24 |
| Valuation of outcomes | 13 | Describe the population and methods used to measure and value outcomes. | Page 8; Line 12 - Line 16 |
| Measurement and valuation of resources  and costs | 14 | Describe how costs were valued. | Page 6; Line 1 - Line 26, Page 7; Line 1 – Line 12 |
| Currency, price date, and conversion | 15 | Report the dates of the estimated resource quantities and unit costs, plus the currency and year of conversion. | Page 4; Line 10 -12 |
| Rationale and  description of model | 16 | If modelling is used, describe in detail and why used. Report if the model is publicly available and where it can be accessed. | Page 5; Line 4 - Line 9  Figure 1 |
| Analytics and assumptions | 17 | Describe any methods for analysing or statistically transforming data, any extrapolation methods, and approaches for validating any model used. | Page 6; Line 21 - Line 24  Page 7; Line 1 - Line 5 |
| Characterizing heterogeneity | 18 | Describe any methods used for estimating how the results of the study vary for sub-groups. | Page 8; Line 1 - Line 6 |
| Characterizing  distributional effects | 19 | Describe how impacts are distributed across different individuals or adjustments made to reflect priority populations. | NA |
| Characterizing uncertainty | 20 | Describe methods to characterize any sources of uncertainty in the analysis. | Page 8; Line 8 - Line 19, |
| Approach to engagement with patients and others affected by the study | 21 | Describe any approaches to engage patients or service recipients, the general public, communities, or stakeholders (e.g., clinicians or payers) in the design of the study. | NA |
| **RESULTS** | | |  |
| Study parameters | 22 | Report all analytic inputs (e.g., values, ranges, references) including uncertainty or distributional assumptions. | Page 8; Line 21 - 26  Page 9;  Page 10; Line 1 – 2  Table 1, Table 2,  Figure 2, Figure 3 |
| Summary of main results | 23 | Report the mean values for the main categories of costs and outcomes of interest and summarise them in the most appropriate overall measure. | Page 8; Line 21 - 26  Page 9; Line 1 - 18  Table 2,  Figure 2 |
| Effect of uncertainty | 24 | Describe how uncertainty about analytic judgments, inputs, or projections affect findings. Report the effect of choice of discount rate and time horizon, if applicable. | Page 9; Line 20 - Line 26,  Page 10; Line 1 - 2,  Figure 3 |
| Effect of engagement with patients and others affected by the study | 25 | Report on any difference patient/service recipient, general public, community, or stakeholder involvement made to the approach or findings of the study | NA |
| **DISCUSSION** | | |  |
| Study findings, limitations, generalizability, and current knowledge | 26 | Report key findings, limitations, ethical or equity considerations not captured, and how these could impact patients, policy, or practice. | Pages 10-14 |
| **OTHER RELEVANT INFORMATION** | | | |
| Source of funding | 27 | Describe how the study was funded and any role of the funder in the identification, design, conduct, and reporting of the analysis | Title page |
| Conflicts of interest | 28 | Report authors conflicts of interest according to journal or International Committee of Medical Journal Editors requirements. | Title page |

Husereau D, Drummond M, Augustovski F, de Bekker-Grob E, Briggs AH, Carswell C, Caulley L, Chaiyakunapruk N, Greenberg D, Loder E, Mauskopf J, Mullins CD, Petrou S, Pwu RF, Staniszewska S; CHEERS 2022 ISPOR Good Research Practices Task Force. Consolidated Health Economic Evaluation Reporting Standards 2022 (CHEERS 2022) Statement: Updated Reporting Guidance for Health Economic Evaluations. BMJ. 2022;376:e067975.

The checklist is Open Access distributed in accordance with the terms of the Creative Commons Attribution (CC BY 4.0) license, which permits others to distribute, remix, adapt and build upon this work, for commercial use, provided the original work is properly cited. See: [http://creativecommons.org/licenses/by/4.0/.](http://creativecommons.org/licenses/by/4.0/)
